# Standardization of Epidemiological Surveillance of Invasive Group A Streptococcal Infections^[Author-notes ofac281-FM1]^

**DOI:** 10.1093/ofid/ofac281

**Published:** 2022-09-15

**Authors:** Kate M Miller, Theresa Lamagni, Thomas Cherian, Jeffrey W Cannon, Tom Parks, Richard A Adegbola, Janessa Pickering, Tim Barnett, Mark E Engel, Laurens Manning, Asha C Bowen, Jonathan R Carapetis, Hannah C Moore, Dylan D Barth, David C Kaslow, Chris A Van Beneden

**Affiliations:** Wesfarmers Centre of Vaccines and Infectious Diseases, Telethon Kids Institute, University of Western Australia, Nedlands, Western Australia; UK Health Security Agency, London, United Kingdom; MMGH Consulting GmBH, Geneva, Switzerland; Wesfarmers Centre of Vaccines and Infectious Diseases, Telethon Kids Institute, University of Western Australia, Nedlands, Western Australia; Department of Global Health and Population, Harvard T.H. Chan School of Public Health, Boston, Massachusetts, USA; Department of Infectious Disease, Imperial College London, Hammersmith Hospital, London, United Kingdom; Nigerian Institute of Medical Research, Lagos, Nigeria; Wesfarmers Centre of Vaccines and Infectious Diseases, Telethon Kids Institute, University of Western Australia, Nedlands, Western Australia; Wesfarmers Centre of Vaccines and Infectious Diseases, Telethon Kids Institute, University of Western Australia, Nedlands, Western Australia; AFROStrep Research Initiative, Department of Medicine, University of Cape Town, Cape Town, South Africa; Wesfarmers Centre of Vaccines and Infectious Diseases, Telethon Kids Institute, University of Western Australia, Nedlands, Western Australia; Infectious Diseases Department, Fiona Stanley Hospital, Perth, Western Australia, Australia; Medical School, University of Western Australia, Perth, Western Australia, Australia; Wesfarmers Centre of Vaccines and Infectious Diseases, Telethon Kids Institute, University of Western Australia, Nedlands, Western Australia; Perth Children’s Hospital, Nedlands, Western Australia; Faculty of Health and Medicine, University of Western Australia, Nedlands, Western Australia; Wesfarmers Centre of Vaccines and Infectious Diseases, Telethon Kids Institute, University of Western Australia, Nedlands, Western Australia; Faculty of Health and Medicine, University of Western Australia, Nedlands, Western Australia; Wesfarmers Centre of Vaccines and Infectious Diseases, Telethon Kids Institute, University of Western Australia, Nedlands, Western Australia; Wesfarmers Centre of Vaccines and Infectious Diseases, Telethon Kids Institute, University of Western Australia, Nedlands, Western Australia; Faculty of Health and Medicine, University of Western Australia, Nedlands, Western Australia; PATH, Seattle, Washington, USA; CDC Foundation, Atlanta, Georgia, USA

**Keywords:** epidemiology, infectious disease, invasive infections, *Streptococcus pyogenes*, surveillance

## Abstract

Invasive group A streptococcal (Strep A) infections occur when *Streptococcus pyogenes*, also known as beta-hemolytic group A *Streptococcus*, invades a normally sterile site in the body. This article provides guidelines for establishing surveillance for invasive Strep A infections. The primary objective of invasive Strep A surveillance is to monitor trends in rates of infection and determine the demographic and clinical characteristics of patients with laboratory-confirmed invasive Strep A infection, the age- and sex-specific incidence in the population of a defined geographic area, trends in risk factors, and the mortality rates and rates of nonfatal sequelae caused by invasive Strep A infections.

This article includes clinical descriptions followed by case definitions, based on clinical and laboratory evidence, and case classifications (confirmed or probable, if applicable) for invasive Strep A infections and for 3 Strep A syndromes: streptococcal toxic shock syndrome, necrotizing fasciitis, and pregnancy-associated Strep A infection.

Considerations of the type of surveillance are also presented, noting that most people who have invasive Strep A infections will present to hospital and that invasive Strep A is a notifiable disease in some countries. Minimal surveillance necessary for invasive Strep A infection is facility-based, passive surveillance. A resource-intensive but more informative approach is active case finding of laboratory-confirmed Strep A invasive infections among a large (eg, state-wide) and well defined population.

Participant eligibility, surveillance population, and additional surveillance components such as the use of *International Classification of Disease* diagnosis codes, follow-up, period of surveillance, seasonality, and sample size are discussed. Finally, the core data elements to be collected on case report forms are presented.

## DISEASE CHARACTERISTICS

Invasive group A streptococcal (Strep A) or *Streptococcus pyogenes* infections occur when the organism invades a normally sterile site in the body. Invasive Strep A infections can manifest as any of several clinical syndromes, including empyema, bacteremic pneumonia, primary bacteremia (Strep A isolated from the blood without another apparent focus of infection), bacteremia in association with skin and soft tissue infection (eg, cellulitis, or infection of a surgical or nonsurgical wound), deep soft-tissue infection (eg, myositis, necrotizing fasciitis, internal body abscess), meningitis, peritonitis, osteomyelitis, and septic arthritis. The release of toxins can lead to further complications such as streptococcal toxic shock syndrome (STSS), a syndrome manifesting as hypotension in combination with evidence of multiple organ failure.

The incidence of invasive Strep A infections is highest among the very young and the elderly. Persons at increased risk for invasive Strep A infections include persons with underlying chronic medical (diabetes, cancer, human immunodeficiency virus infection, chronic lung or heart disease) or immunocompromising conditions, indigenous populations (eg, Native Americans, Aboriginals), and persons who experience homelessness or with drug or alcohol addiction [1]. Invasive Strep A infections associated with pregnancy are also an important cause of maternal and infant mortality. Also called “maternal infections”, pregnancy-associated invasive Strep A infections are those associated with pregnancy, delivery, and the postpartum period (or “puerperium”). Invasive Strep A infection usually occurs sporadically; however, outbreaks of invasive Strep A infections occur in crowded, congregate settings, including among residents of long-term care facilities and patients in inpatient settings [2].

Diagnosis of invasive Strep A infections is established via microbiological confirmation of Strep A collected from a normally sterile site. A normally sterile site contains no microorganisms in a healthy individual and includes, but is not limited to, the following: blood, cerebrospinal fluid, pleural fluid, peritoneal fluid, pericardial fluid, bone, muscle, joint fluid, and internal organs such as brain, heart, liver, spleen, or lymph node. Confirmatory microbiological tests include bacterial culture or detection of Strep A by nucleic acid testing. Prognosis varies depending on the type of invasive Strep A infection, and accurate diagnosis and prompt treatment is critical to improve outcomes. Even with aggressive antimicrobial treatment, case fatality remains high globally (∼20%) for invasive Strep A infections, particularly in neonates and older adults and patients with concomitant necrotizing fasciitis or STSS [3, 4].

## OBJECTIVES OF SURVEILLANCE FOR INVASIVE GROUP A STREPTOCOCCAL INFECTIONS

An effective surveillance system for invasive Strep A serves to monitor trends in the following (1) numbers or incidence rates of laboratory-confirmed invasive Strep A infection for a defined population; (2) demographic and clinical characteristics of patients; (3) age- and sex-specific incidence of invasive infections; and (4) incidence rates of death and nonfatal sequelae due to invasive Strep A infections.

Through monitoring trends in these factors, changes warranting initiation of rapid or longer term public health measures can be implemented, including in response to outbreaks.

Enhanced surveillance systems may also aim to determine and monitor the distribution of select genotypic or phenotypic features (ie, *emm* types, virulence factors, presence of vaccine antigens, and antimicrobial susceptibility) of Strep A strains to (1) measure strain-specific disease burden and facilitate outbreak detection; (2) monitor trends in Strep A strains causing invasive disease to detect shifts in predominant or virulent strains over time, including shifts due to strain-specific vaccination; (3) track antimicrobial resistance over time; and (4) predict and evaluate the effectiveness of future or existing strain-specific vaccines.

Potential additional surveillance objectives (these objectives are optional and not required in every surveillance system) may include the following: (1) monitor the impact of interventions and public health policies on rates of invasive Strep A infections, including efforts to reduce secondary spread in households; (2) identify potentially modifiable risk factors for community-acquired invasive Strep A infections; and (3) identify and monitor potentially preventable invasive Strep A infections, such as healthcare-associated infections (eg, postpartum, postsurgical, or those occurring in nursing homes) or invasive infections associated with facilities such as homeless shelters.

## CASE DEFINITIONS AND CASE CLASSIFICATION

Standardized case definitions are important for obtaining accurate surveillance data, comparing burden estimates and case fatality rates across surveillance sites, and monitoring the impact of vaccines and other interventions. The case definitions for invasive Strep A infections in Table 1 have been adapted from the US Centers for Disease Control and Prevention (CDC)’s Active Bacterial Core surveillance (which established uniform criteria for reporting cases of invasive Strep A infections) and the Working Group on Severe Streptococcal Infections, which established a case definition for STSS [5, 6]. Definitions of necrotizing fasciitis, a severe manifestation of invasive Strep A infection and invasive Strep A peripartum infections, are also provided below in Tables 2 and 3, respectively.

**Table 1.** Case Definitions and Classifications of Invasive Strep A Infections for Surveillance

***Probable case***
A case of probable invasive Strep A infection is defined as a clinically severe illness, such as maternal sepsis, septic shock, STSS, or necrotizing fasciitis, for which no other bacterial etiology has been identified and in which Strep A is isolated or detected from a nonsterile site (eg, throat, sputum, wound, superficial skin abscess, subcutaneous tissue, or placenta)^a^. For surveillance purposes, infections in women who develop clinical signs of postpartum endometritis and Strep A is isolated from the cervix (a nonsterile site) should be included as probable cases.
***Confirmed case***
A case of confirmed invasive Strep A infection is defined as an illness associated with isolation of Strep A (*Streptococcus pyogenes*) by culture or detection of Strep A by nucleic acid testing from a normally sterile site (eg, blood, cerebrospinal fluid, joint fluid, peritoneal fluid, bone, internal organs).

a This is a modification of CDC’s ABCs case definition that would categorize STSS and necrotizing fasciitis in association with Strep A cultured from a wound as a confirmed invasive infection.

**Table 2.** Case Definitions and Classification of Necrotizing Fasciitis

*Probable case:* A probable case of Strep A necrotizing fasciitis requires both clinical evidence and suggestive laboratory evidence.
Clinical evidence: Gross fascial edema and necrosis detected at surgery and/or Necrosis of superficial fascia and polymorphonuclear infiltrate and edema of the reticular dermis, subcutaneous fat, and superficial fascia detected by histopathology.
Suggestive laboratory evidence: Isolation by culture or detection of Strep A by nucleic acid testing of a specimen obtained from a nonsterile site (eg, throat, sputum, wound, superficial skin abscess, subcutaneous tissue, or placenta).
*Confirmed case:* A confirmed case of Strep A necrotizing fasciitis requires both clinical evidence (see above) and definitive laboratory evidence.
Definitive laboratory evidence: Strep A isolated by culture or Strep A detected by nucleic acid testing from a specimen obtained from a normally sterile site (eg, blood, muscle, fascia).

**Table 3.** Case Definitions and Classifications of Invasive Strep A Peripartum Infections for Surveillance

*Probable case:* A probable case of invasive peripartum infection due to Strep A is defined as a pregnancy-associated infection that occurs in the peripartum period (intrapartum or postpartum) and for which Strep A is cultured from a typically nonsterile site (eg, swab of the oropharynx, an endometrial aspirate, urine culture) and no other organisms are identified as the etiology of the infection.
*Confirmed case:* A confirmed case of peripartum infections due to Strep A is defined as a pregnancy-associated infection that occurs in the peripartum period and for which Strep A is cultured from a normally sterile site.

### Notes About Case Definitions

The inclusion of probable cases with Strep A cultured from nonsterile sites requires an accurate clinical diagnosis. Not all sites will have this capacity, nor will all studies include cases from nonsterile sites; thus, the numbers or incidence rates of confirmed and probable invasive Strep A infections should be presented separately. This will identify a core set of confirmed cases and allow for comparisons between studies.

A list of normally sterile sites is provided in Supplementary Appendix 1. Isolates obtained from the pharynx, trachea, or bronchi should not be considered isolates from a normally sterile site because these are contiguous with the throat and nonsterile. Recovery of Strep A from these sites may represent colonization. However, Strep A isolated from bronchoalveolar lavage in a patient with a clinical diagnosis of pneumonia may be considered a confirmed case of invasive Strep A infection.

### Streptococcal Toxic Shock Syndrome

Streptococcal toxic shock syndrome (STSS) is a severe illness associated with Strep A infection, manifesting with shock and multisystem organ failure, and most often occurs in association with skin and soft-tissue infections but can occur in association with any Strep A infection [4, 7].

Criteria for diagnosing confirmed and probable STSS have been defined by The Working Group on Severe Streptococcal Infections and Council of State and Territorial Epidemiologists [6, 8]. The standardized working group definition of STSS is detailed in Supplementary Appendix 2. However, an STSS diagnosis should be considered for all patients in septic shock from whom Strep A is isolated.

### Necrotizing Fasciitis

Necrotizing fasciitis is a rapidly progressive infection that destroys deep soft tissues, including muscle fascia and overlying subcutaneous fat. Multiple species of bacteria can cause necrotizing fasciitis; however, Strep A is a common cause of this syndrome. Imaging (computed tomography, magnetic resonance imaging, ultrasound, and radiography) can reveal the presence of swelling, inflammation, and gas in soft tissue; however, it cannot be relied on, or should not be used alone, for diagnosis without evidence of necrotizing fasciitis by visual examination of tissue during surgery or by histopathologic examination. Diagnostic tests should not delay definitive surgical management, because delays can lead to increased morbidity and mortality. Case definitions and classification are summarized in Table 2.

### Pregnancy-Associated Infections

Invasive Strep A infections associated with pregnancy are an important cause of maternal and infant mortality. Also called “maternal infections”, pregnancy-associated invasive Strep A infections are those associated with pregnancy, delivery, and the postpartum period (or “puerperium”), although most infections occur in the postpartum period [9]. The postpartum period is typically defined as beginning with the delivery of the infant and extending to 6–12 weeks after delivery, when most physiologic systems have returned to the prepregnancy state [10]. Pregnancy-associated infections include infections of the uterus, vagina, external genitalia, as well nongenital focal infections such as bacteremic pneumonia and septic arthritis. Strep A can invade the endometrium and adjacent structures, lymphatics, and bloodstream and can result in systemic infections, such as STSS. Postpartum (or puerperal) sepsis may result from maternal colonization with Strep A, most commonly from the pharynx, or transmission from close contacts including healthcare staff. Pregnancy-associated definitions and invasive Strep A definitions are not necessarily mutually exclusive; pregnancy-associated definitions incorporate timing of disease onset relative to childbirth.

An important subset of pregnancy-associated infections is peripartum infections. The term “peripartum infection” accounts for both intrapartum (intra-amniotic infection occurring before birth) and postpartum (or puerperal) bacterial infections related to childbirth. Peripartum infection due to Strep A includes the following: infection of the genitourinary systems related to labor, delivery, and the puerperium; infections specifically related to the birth process but not of the genitourinary systems (eg, breast abscess, surgical site infection); and incidental infections (eg, bacteremic pneumonia), occurring at any time between the onset of membrane rupture or labor and the 42^nd^-day postpartum and for which Strep A is the etiology of the infection [11, 12]. Case definitions and classification are provided in Table 3.

### Other Surveillance Definitions

Some surveillance systems may record the mode of transmission of invasive Strep A infections if it is known. Monitoring sources of disease transmission is essential in calculating and comparing attack rates and identifying outbreaks and at-risk populations or settings. Data can also be used to inform control and prevention strategies. The following definitions are suggested for classifying modes of transmission: (1) healthcare-associated infection - an infection that occurs in a person whose first positive specimen was collected >48 hours after hospital admission and <7 days postdischarge [13]; (2) secondary transmission - an invasive Strep A infection that occurs in a person who had contact with (was epidemiologically linked to) another person who had an infectious Strep A case within the preceding 30 days [13]; and (3) community transmission - if not identified as healthcare-associated or secondary transmission, it is assumed that Strep A had been acquired in the community [14].

## SPECIMEN COLLECTION AND DETECTION OF STREP A

### Specimen Collection

Collection of specimens from normally sterile sites should be performed in a standardized fashion. Appropriate specimens include swabs (eg, from wounds or fasciitis sites), blood from venipuncture, aspirated body fluid or purulent material, or tissue (eg, biopsy material from fasciitis site). Ideally, for patients with suspected invasive Strep A infection, blood cultures (2 sets) should be obtained before antibiotic administration. Personnel should be trained in the performance of the sterile procedures required (ie, blood culture, lumbar puncture, joint fluid aspiration, pleural fluid obtained by thoracentesis, etc), and a standard operating procedure for obtaining, processing, transporting, and storing isolates should be developed and followed. Proper technique will increase the yield of normally sterile site cultures and reduce the growth of skin contaminants. If such training and practice is not feasible, investigators will rely on local clinical practices.

Although collection of specimens from normally sterile sites is preferred, probable cases of invasive Strep A infection can be identified by collection of a specimen obtained from a nonsterile clinically relevant site such as throat, sputum, wound, superficial skin abscess, subcutaneous tissue, or placenta. For patients with skin or soft tissue infection, a wound culture should be obtained. Patients who undergo debridement should have debrided material sent for culture. Postpartum women should have endometrial aspiration for Gram stain and culture. Patients with pneumonia should have throat and sputum culture. See Table 4 for a list of the typical body sites from which Strep A is cultured by type of invasive infection (clinical syndrome).

**Table 4.** Recommended Specimens to Collect for Diagnosis of Invasive Strep A Syndromes

Type of Invasive Infection	Typical Body Sites From Which Strep A Is Cultured
Necrotizing fasciitis	Wound and blood
Streptococcal toxic shock syndrome	Blood, throat, and wound, if appropriate
Septic shock	Blood
Puerperal sepsis	Blood and endometrium culture, if appropriate
Septic arthritis	Blood and sterilely obtained joint fluid
Streptococcal meningitis	Blood, cerebrospinal fluid
Cellulitis	Blood, sterilely obtained tissue aspirate/specimen
Lymphangitis	Blood, sterilely obtained lymph node aspirate/specimen
Osteomyelitis	Blood, sterilely obtained bone aspirate/specimen
Empyema or pneumonia with effusion	Blood, sterilely obtained pleural fluid
Pneumonia	Blood, lung specimen (taken via bronchoalveolar lavage, bronchoscopy, or open lung biopsy), and pleural fluid
Bacteremia	Blood
Sepsis or septicemia	Blood

Abbreviations: Strep A, group A streptococcal.

### Specimen Transport

Aspiration into a syringe is the standard method of sampling body fluid collected from a normally sterile site, abscess fluid, or other localized Strep A infection. Biopsy material may also be obtained in the diagnosis or treatment of necrotizing fasciitis. All fluid and tissue specimens should be immediately transported to the laboratory in a sterile container, but never with a needle attached. Tissue specimens must be kept moist by adding a few drops of sterile, non-bacteriostatic saline to the container or by placing the sample on a sterile piece of moistened gauze. Fluid or pus may also be sent on swabs and transported to a microbiology laboratory. See Supplementary Appendix 3 for further detail regarding specimen storage, documentation, and transfer.

### Detection of Strep A

Bacterial culture is currently considered the gold standard for detection of Strep A as the etiology of invasive infections [15]. However, point-of-care nucleic acid amplification tests (NAATs) may become an important option for surveillance studies in some resource-poor settings and/or locations where microbiology laboratories are not available [16, 17]. Bacterial culture is necessary to obtain the Strep A isolate if further characterization is part of the surveillance program. Molecular profiling of *emm* types (via the CDC methodology [18] or whole-genome sequencing [WGS]-derived methods [19]), or WGS to differentiate Strep A strains, can support surveillance by indicating the diversity of strains in a population over time, and to map transmission in communities.

Routine microbiological culture of blood or other specimens is performed in a laboratory setting with appropriate quality control. To limit potential contamination of blood cultures during sampling, sterile gloves must be used, and blood should be drawn from a vein through skin that has been precleaned with an antiseptic (eg, alcohol iodine-containing preparation or 70% alcohol wipe) [20]. Sterile blood culture bottles should be used for blood collection and transport to a laboratory for subsequent bacterial identification. Traditional blood cultures require static incubation at 37°C and visual inspection of bacterial growth by a trained laboratory worker; however, this can take up to 48 hours and the microorganism requires subsequent identification (eg, agar plate culture and confirmation as below) [21]. Modern blood culture methods may use more automated methods to identify bacterial growth and detect pathogens. Direct detection of Strep A from blood culture specimens can be performed rapidly and reliably using NAATs, including US Food and Drug Administration-approved molecular panels, greatly shortening the time for medical intervention (eg, administration of antibiotics) [15]. Modern molecular detection technologies will continue to emerge and reduce the time to pathogen identification.

Agar plates inoculated with blood cultures or other clinical samples are initially incubated at 37°C for 18–24 hours, but incubation up to 48 hours may be necessary. The addition of 5%–10% CO_2_ for incubation may enhance growth but is not essential. After incubation, plates are inspected for β-hemolytic colonies to undergo subculture purification and confirmation with further biochemical tests including latex agglutination testing (for Lancefield groups A, C, G), bacitracin sensitivity, and pyrrolidonyl arylamidase testing. No biochemical test is 100% specific for *S pyogenes* [15] and so they are frequently used in combination. Purified colonies can be stored to enable further testing, with long-term storage between −70 and −80°C in a suitable cryoprotectant medium [eg, in Todd Hewitt Glycerol broth or skim milk tryptone glucose glycerol broth (STGGB)].

### Strain Identification and Characterization

Group A *Streptococcus* strains have been traditionally typed based on antigenic variation of the M protein (M typing) and major pilus subunit protein (T typing). Historically, these were determined by serology, but deoxyribonucleic acid sequence analysis of the variable region of the *emm* gene (*emm* typing) is now preferred [18, 22]. *Emm* types can also be categorized as *emm* clusters, based on bioinformatic criteria [23].

Although this provides a general overview of Strep A complexity within a given population, it does not resolve individual strains. Strain identification requires additional molecular approaches that include multilocus sequence typing ([MLST] based on sequence determination of a core set of housekeeping genes) and, more commonly, WGS of individual strains. More importantly, each of the classic typing schemes (eg, *emm* type, MLST) can be determined from WGS data, and the diminishing costs and unprecedented resolution of WGS make this the preferred method for strain identification. WGS data also provide information on virulence genes, vaccine antigens, transmission, and antibiotic resistance gene carriage.

### Antimicrobial Susceptibility Testing

Penicillin remains the antibiotic treatment of choice for most Strep A infections. However, because approximately 10% of persons have an allergy to penicillin, second-line antibiotic treatments include macrolides (eg, erythromycin, clarithromycin, azithromycin) and lincosamides (eg, clindamycin). Clindamycin, used as an adjunctive therapy with penicillin, is recommended for severe invasive Strep A infections, such as necrotizing fasciitis and STSS [24].

At this time, Strep A is universally sensitive to penicillin and other β-lactam antibiotics such as cephalosporins. However, mutations in penicillin-binding protein genes that confer reduced susceptibility to β-lactam antibiotics have been reported [25, 26]. Resistance to macrolides and to clindamycin changes by time and geographic location; during periods of increased resistance, nonsusceptibility to macrolides or clindamycin can be detected in >20% of invasive Strep A infections in some communities [24]. Resistance to clindamycin may be inducible or constitutive. Susceptibility testing for both penicillin and clindamycin is therefore recommended. Some investigators may choose to monitor nonsusceptibility to fluoroquinolones and tetracycline. Susceptibility testing to trimethoprim/sulfamethoxazole, ciprofloxacin, and macrolides (ie, erythromycin, azithromycin) may also be included because these antibiotics are often used for treatment of noninvasive Strep A infections (eg, pharyngitis) or for treatment of *Staphylococcus aureus* (methicillin-resistant *S aureus*), an important bacterial cause of skin infections and toxic shock syndrome.

Antimicrobial susceptibility testing is usually determined phenotypically by measuring minimum inhibitory concentrations for individual antibiotics. Methods to determine this include disk diffusion, Etest, and broth microdilution, following standardized protocols published by the European Committee on Antimicrobial Susceptibility Testing (EUCAST) [27] and the Clinical and Laboratory Standards Institute [28]. Molecular techniques (eg, polymerase chain reaction, WGS) can be used to obtain information on antibiotic resistance gene carriage by individual Strep A strains. However, caution should be exercised in directly correlating antibiotic resistance gene carriage with phenotypic susceptibility, because this direct link has not always been demonstrated experimentally [29].

## CASE ASCERTAINMENT AND SURVEILLANCE SETTINGS

Invasive Strep A infections are typically acute and severe; many are life-threatening. In contrast to less severe manifestations of Strep A infections such as pharyngitis and impetigo, most people with an invasive Strep A infection will seek medical care. Therefore, the surveillance approach to identifying cases among people who present for medical care may be either passive or active (Supplementary Appendix 4).

In some countries, invasive Strep A infections are notifiable, meaning probable or confirmed cases are required by law to be immediately reported to local or state health authorities. If invasive Strep A infections are notifiable within the surveillance jurisdiction, healthcare providers should be engaged with, and regularly reminded of, their responsibility to report suspected cases.

Consideration needs to be given to establishing surveillance across sites to capture the full array of patients. Because isolation or detection of Strep A from a normally sterile site is essential to the definition of a confirmed case of invasive disease, the microbiology laboratories in acute care hospitals and reference laboratories processing specimens for residents of the surveillance area are efficient sites for case identification. Where possible, laboratory data should be further augmented through review or data linkage to medical records such as admission and discharge registers, medical charts, and operating room records or death certificates for clinical syndromes typical of invasive Strep A (eg, necrotizing fasciitis, STSS). Medical records providing clinical diagnosis are essential sources of information if the surveillance protocol includes probable cases with Strep A isolates from nonsterile sites. Note that relying solely on medical records can be unreliable to estimate the incidence of invasive Strep A infections because cases can be misdiagnosed and the clinical information or causative pathogen needed to confirm a case of invasive Strep A infection may not be recorded. Considerations for using administrative health databases to identify cases are provided in Supplementary Appendix 5. Private hospitals and outpatient facilities are also potential data sources; however, they should not be used in isolation because they may only represent a small fraction of all invasive Strep A infection cases.

Surveillance for STSS can be conducted by requiring that the illness meets the criteria defined in Supplementary Appendix 2, ascertained by reviewing the medical chart and laboratory values for all cases of invasive Strep A that result in hypotension and are suspected to be STSS. Alternatively, surveillance for STSS can rely on provider diagnosis of STSS as indicated in the medical chart (eg, provider notes, hospital discharge diagnosis). The method chosen should be clearly stated in the description of the surveillance system and data summaries. Note that there is currently no *International Classification of Disease* (ICD) diagnosis code for STSS.

For each data source, surveillance staff should (1) know the purpose of the data source (ie, routinely collected as part of patient care, mandatory collection of data under legal mandates, collected for research purposes, other), (2) identify any legal mandates governing the operations of the data source that may impact the accessibility or quality of the data from that source, and (3) describe the representative population for the data.

## TYPES OF SURVEILLANCE

The selection of surveillance strategies depends on specific epidemiologic and clinical characteristics of the disease outcome of interest, the overall surveillance objectives, surveillance location, services accessibility, and the resources available (see Supplementary Appendix 6 for surveillance definitions). In low- and middle-income countries, the resources needed to implement active surveillance and laboratory confirmation may not be available.

A quality management plan should be written before the start of surveillance to establish and ensure the quality of processes, data, and documentation associated with surveillance activities. All surveillance should be conducted in accordance with ethical guidelines (see Supplementary Appendix 7). Minimal and enhanced surveillance strategies for invasive Strep A infections are described in Table 5 to provide guidance for those with limited resources and those with greater capacity, respectively.

**Table 5.** Strategies for Surveillance of Invasive Strep A Infections

*Minimum Surveillance*
The minimum surveillance for invasive Strep A infection is facility-based, passive surveillance. Passive surveillance is based on clinical signs, symptoms and a diagnosis recorded in health facility databases, and microbiological data from laboratory databases. Minimal surveillance may be adequate if the health facility protocol ensures that blood cultures are collected routinely from hospitalized, febrile patients and those with signs and symptoms consistent with invasive Strep A infections as part of routine clinical evaluation. In settings with limited resources, surveillance for invasive Strep A infection may be limited to sentinel hospitals. Where possible, healthcare utilization surveys should be performed periodically to estimate the size of the catchment population seeking care at the sentinel hospital, which will enable the estimation of a population denominator for incidence calculations [30–32]. Reporting sources, which include microbiologists, laboratory scientists, clinicians, and infection control practitioners, can be instructed to report all cases of invasive Strep A infections to the surveillance team. Standard case report forms may be provided to the health facilities or laboratories to encourage completion and submission to the surveillance program.
*Enhanced Surveillance*
Enhanced surveillance results in a more precise estimation of age-specific disease and fatality rates than other surveillance methods. Enhanced surveillance for invasive Strep A infection is prospective, active, facility-based surveillance. Active case finding of laboratory-confirmed Strep A invasive infections is best implemented in 1 or more healthcare facilities providing care to a large (eg, statewide) and well-defined population. Timely detection of new cases ensures that case investigations and data abstractions from laboratory and medical records are complete [33]. Before starting surveillance, well-defined clinical practices and laboratory methods should be established and remain constant throughout the surveillance period. Efforts to identify all cases among persons who live within the surveillance catchment area and exclude cases identified in persons who seek care at hospitals within the surveillance catchment area but live outside the catchment area are important for appropriate matching of numerator and denominators and for calculating disease incidence. Active surveillance maximizes case ascertainment and data collection through review of a line listing of potential cases from clinical and laboratory reports from emergency department databases, hospital admission or discharge log databases, outpatient clinics or laboratories. Where hospital and microbiological data are computerized, surveillance personnel routinely obtain electronic line listings of all probable cases and positive Strep A laboratory test. Where data are not computerized, surveillance staff regularly liaise with hospital medical staff and intensive care, and routinely review the relevant and available laboratory results to identify any new patients with invasive Strep A infections. Audits should be performed biannually to assess the completeness of case ascertainment, accuracy of data collected, timeliness, and laboratory performance.

## SURVEILLANCE POPULATION

Surveillance protocol should clearly describe enrollment eligibility criteria. Persons with underlying immunocompromising conditions or, chronic diseases, or pregnant or lactating women should not be excluded from surveillance.

The surveillance population includes all residents of the laboratory or hospital catchment area. The surveillance will typically occur in a defined geographic or medical facility catchment area served by the laboratory performing the cultures, and therefore the denominator must be defined as the total number of eligible at-risk people. This population, or denominator, must be properly characterized a priori to derive meaningful disease burden estimates. Without an accurate account of all people in the population who could potentially be evaluated for invasive Strep A, disease estimates may be under- or overestimated [30, 31].

Because invasive Strep A infections are relatively rare in most populations, it is preferable to conduct surveillance across a large population to maximize the number of cases ascertained. This minimizes the confidence intervals around the point estimate of disease incidence. However, large populations are usually not well defined through demographic surveillance. Data accuracy must be assured if government-derived census data are used to determine the community’s demographic profile, such as the number of people in relevant age categories. Ongoing demographic surveillance might be necessary to generate reliable burden estimates if surveillance extends over a long period of time or if a population is not stable because of mobility or other logistic factors. This is a particular challenge if the population is covered by numerous hospitals, or if there is a substantial likelihood that cases occurring within the surveillance region may attend a tertiary or specialist hospital outside the surveillance region. Cases occurring in people residing outside the defined catchment area should be excluded.

When surveillance is based in a sentinel hospital and is not population-based, healthcare utilization surveys can be used to determine those accessing healthcare to estimate the population served, or the denominator, that corresponds to the cases of interest [32, 34]. The denominator is the number of patients within the geographical catchment area who would be expected to attend that hospital if signs and symptoms of invasive Strep A infections develop.

## SPECIAL CONSIDERATIONS FOR INVASIVE STREP A SURVEILLANCE

### Characterization of Invasive Strep A Isolates

Some invasive Strep A surveillance includes monitoring and describing the distribution of select genotypic or phenotypic features of Strep A isolates (eg, *emm* types, presence of vaccine antigens, or antimicrobial susceptibility). The objectives of these surveillance systems can include estimating strain-specific disease burden, evaluating the effectiveness of prospective or existing strain-specific vaccines, detecting shifts in predominant or virulent strains over time, and tracking antimicrobial resistance [35].

In many hospitals and laboratories, bacterial cultures grown from clinical specimens are not stored once a pathogen has been detected. For invasive Strep A surveillance systems, which also seek to describe select genotypic or phenotypic features of Strep A strains, the associated laboratories should be reminded to store all Strep A isolates for a minimum time (eg, >6 months) to allow for additional characterization and for potential strain comparisons in outbreak investigations [36]. Acquisition of additional freezer space at the laboratories or identification of a reference laboratory where cultures can be sent and stored may be needed.

### Antimicrobial Resistance

Antimicrobial resistance among invasive Strep A isolates is typically expressed as the proportion or prevalence of isolates demonstrating resistance to the antibiotic. Monitoring local antimicrobial resistance and trends over time is important for local healthcare providers making treatment plans for patients and public health agencies. When calculating the prevalence of antimicrobial resistance, it is important to avoid double-counting results from testing more than 1 isolate per patient per infection. In addition, the percentage of isolates used for antimicrobial testing should be calculated and included in any descriptions of antimicrobial resistance.

### Active Follow-up of Cases

The specific protocol will determine the extent of follow-up of patient illness outcomes. Data collected on invasive Strep A infections should include in-hospital mortality or mortality within 30 days of illness onset and a range of other severe outcomes such as renal failure and major and minor amputation, where possible. Data on clinical outcomes can be obtained via follow-up surveys conducted several months post discharge, with the patient’s consent.

### Microbiological Sampling of Suspect Cases

Hospitals that obtain bacterial cultures as part of routine practice should ensure that appropriate cultures are obtained prior or close to initiation of antibiotic treatment of patients with the following syndromes: sepsis and septic shock, necrotizing fasciitis, puerperal sepsis, arthritis or septic joint, meningitis, erysipelas, lymphangitis, and osteomyelitis. Any protocol that results in changes to clinical practice would need to occur before starting a prospective study because such changes will likely alter case detection.

### *International Classification of Diseases* Diagnosis Codes

*International Classification of Diseases* diagnosis codes available in the country of surveillance can be used to identify several invasive Strep A infections. However, for many invasive Strep A infections, ICD diagnosis codes are unreliable. For some syndromes, a specific code for the infection does not exist (eg, STSS) or is not specific for Strep A as the cause of the syndrome (eg, necrotizing fasciitis, osteomyelitis) (see Supplementary Appendix 8). In addition, a diagnosis code assigned to a patient may be based on a clinical assumption of a specific Strep A infection without being confirmed by laboratory testing; many disease syndromes may be caused by multiple pathogens. The positive predictive value of ICD codes for many invasive Strep A infections is therefore often poor. Understanding local practices in coding is essential to interpreting and using ICD codes. If surveillance relies on ICD diagnosis codes, an additional Strep A-specific code may be required to classify the infection as a confirmed invasive Strep A case. Care should be taken to note any subtle differences between the international versions because ICD diagnosis codes may be different.

### Frequency

Case ascertainment can be improved if the surveillance team makes regular visits to the surveillance sites to review clinical and laboratory records for missed cases and inform healthcare workers of the goals and methods of Strep A surveillance.

### Period of Surveillance

The duration of surveillance depends on the availability of resources to support the surveillance system and the time needed to achieve the surveillance objectives. Multiple years of surveillance are generally required to detect outbreaks, to evaluate temporal trends (eg, changes in demographics and risk factors of the underlying population, changing etiologic Strep A strain characteristics) or the impact of an intervention such as the introduction of a vaccine program.

### Season

Investigators should consider conducting surveillance through a full year to include all months or seasons. In temperate climates, invasive Strep A infections often exhibit seasonal peaks (typically during winter or cooler months) and troughs (typically during summer months). This is not the case in all climates; however, completion of surveillance over a 12-month period enables capture of other nonseasonal cyclic variations. Several years of surveillance is necessary to describe seasonality.

### Sample Size

To derive meaningful estimates of disease burden, the size of the population under surveillance should be considered, given the relatively low incidence of invasive Strep A disease compared with other acute Strep A infections.

### Measurement of Disease Burden

It is important to record each case of invasive Strep A infection, but not to record any case more than once during a single hospitalization or illness episode, defined as within 30 days of the first positive culture result. For example, if a patient has an illness during which Strep A grows from multiple body sites (eg, blood and pericardial fluid) correlating with 2 diagnoses (eg pericarditis and bacteremia) or a patient is diagnosed with both necrotizing fasciitis and STSS due to Strep A, his/her illness should be counted as a single case of invasive Strep A infection.

Given the frequency of invasive Strep A infections, incidence is typically expressed as cases per 100 000 population per year. Incidence rates for confirmed and probable episodes should be reported separately. It is also helpful for regional comparison if reported incidence rates are stratified by the body site from which Strep A was cultured (eg, incidence of invasive infections where Strep A was isolated from the blood), because many healthcare facilities do not routinely culture some body sites (eg, pleural fluid, bone, joint fluid) for which Strep A infection is suspected. Decisions on enumerating these episodes should be made at the analytical level.

## DATA COLLECTION, REPORTING, AND USE

Case report forms should be based on collecting only the information required to achieve the surveillance objectives. See Supplementary Appendix 9 for a list of recommended and optional variables for inclusion in all case report forms.

“General surveillance variables” include unique identifier, date and time of first enrollment or specimen collection, and site where participant is seen, such as setting, location, postcode, state/province/region, country. Each encounter should also record a surveillance visit number/episode number if repeated episodes from the same person are included.

“Key demographic variables” include date of birth or age (in days or months if <12 months and otherwise in years), sex, ethnic origin/race, residential postcode, state, and country, and residential setting at the time of infection (ie, private residence, long-term care/skilled nursing facility, acute care hospital, long-term acute care hospital, incarcerated, homeless).

“Clinical and epidemiologic variables” include site of Strep A infection, clinical risk factors, severity of illness and disease outcome, potential portal of Strep A entry, epidemiologic risk factors, treatment details, and microbiological variables.

## Supplementary Data

Supplementary materials are available at *Open Forum Infectious Diseases* online. Consisting of data provided by the authors to benefit the reader, the posted materials are not copyedited and are the sole responsibility of the authors, so questions or comments should be addressed to the corresponding author.

## Supplementary Material

ofac281_Supplementary_Data

## Notes

***Financial support*.** This manuscript is funded by The Wellcome Trust, Grant Agreement Number 215490/Z/19/Z.

***Supplement sponsorship*.** This supplement is sponsored by the Telethon Kids Institute with additional support from the Wellcome Trust.
