# Supplementary material for: Standardization of Epidemiological Surveillance of Invasive Group A Streptococcal Infections
Source: Open Forum Infect Dis. 2022 Sep 15;9(Suppl 1):S31–40. doi: 10.1093/ofid/ofac281 (PMC9474937; doi:10.1093/ofid/ofac281)
Supplement: ofac281_Supplementary_Data [file ofac281_supplementary_data.docx]

**Standardization of Epidemiological Surveillance of Invasive Group A Streptococcal Infections**

Supplementary Appendices

Table of Contents

[Appendix 1: Normally Sterile Sites 2](#_Toc112321278)

[Appendix 2: Diagnostic Criteria for Streptococcal Toxic Shock Syndrome 3](#_Toc112321279)

[Appendix 3: Specimen Storage, Documentation, and Transfer 4](#_Toc112321280)

[Appendix 4: Comparisons of Advantages and Disadvantages of Active and Passive Surveillance 5](#_Toc112321281)

[Appendix 5: Administrative Health Databases 6](#_Toc112321282)

[Appendix 6: Definitions of Key Surveillance Terms 7](#_Toc112321283)

[Appendix 7: Good Practice and Ethical Considerations 8](#_Toc112321284)

[Appendix 8: Invasive Strep A Infections: Clinical Syndromes and Related ICD-10 codes 9](#_Toc112321285)

[Appendix 9: Variables to be Included in Invasive Strep A Surveillance Datasets 11](#_Toc112321286)

[References 15](#_Toc112321287)

## Appendix 1: Normally Sterile Sites

Normally, sterile sites are those in which bacteria are not present in a healthy person. Below is a list of the common normally sterile sites (and exceptions).

Examples of normally sterile sites:

- blood
- cerebrospinal fluid (CSF)
- pleural fluid
  - - chest fluid
    - thoracentesis fluid
- peritoneal fluid
  - includes:
    - abdominal fluid
    - ascites
- pericardial fluid
- bone
  - includes:
    - bone marrow
    - synovial tissue
- joint fluid
  - includes:
    - synovial fluid
    - fluid, needle aspirate, or culture of any the following joints: knee, ankle, elbow, hip wrist
- internal body sites
  - specimen obtained from surgery or aspirate from one of the following:
    - lymph node
    - brain
    - heart
    - liver
    - muscle
    - spleen
    - vitreous fluid
    - kidney
    - pancreas
    - ovary
    - vascular tissue

## Appendix 2: Diagnostic Criteria for Streptococcal Toxic Shock Syndrome^1^

| **Streptococcal toxic shock syndrome – case definition** |
| --- |
| 1. Isolation of Strep A 2. From sterile site 3. From non-sterile site |
| 1. Clinical signs of severity 2. Hypotension defined by a systolic blood pressure ≤90 mm Hg for adults or less than the fifth percentile by age for children aged <16 years, AND 3. Multi-organ involvement, characterized by two or more of the following:    1. *Fever:* (>38.5°C)    2. *Renal impairment*: Creatinine ≥2 mg/dL (≥177 µmol/L) for adults or greater than or equal to twice the upper limit of normal for age. In patients with pre-existing renal disease, a greater than two-fold elevation over the baseline level.    3. *Coagulopathy:* (platelets ≤100,000/mm^3^ or disseminated intravascular coagulation), defined by prolonged clotting times, low fibrinogen level, and the presence of fibrin degradation products.    4. *Liver involvement*: Alanine aminotransferase, aspartate aminotransferase, or total bilirubin levels more than twice the upper limit of normal for the patient’s age. In patients with pre-existing liver disease, a greater than two-fold increase over the baseline level.    5. *Acute respiratory distress syndrome*: defined by acute onset of diffuse pulmonary infiltrates and hypoxemia in the absence of cardiac failure or by evidence of diffuse capillary leak manifested by acute onset of generalized edema, or pleural or peritoneal effusions with hypoalbuminemia.    6. *Extensive tissue necrosis:* including necrotizing fasciitis or myositis, or gangrene.    7. A generalized erythematous macular *rash* that may result in desquamation |
| A confirmed case is defined as 1A + 2(A+B)  A probable case is defined as 1B + 2(A+B) |

## Appendix 3: Specimen Storage, Documentation, and Transfer

*Storage and handling*

- All specimens should be stored in sealed biohazard plastic bags or inside a biohazard labelled sealed container
  - Store at the temperature required by culture medium. For example, room temperature storage is suitable for eSwabs (Copan, Italy), whereas refrigerated (in fridge) conditions are recommended for specimens stored in STGGB
- Sample collection documentation must be kept with specimens, but not in the same compartment in case of leakage

*Documentation*

- Label all specimens
  - Follow instructions on sticky label on tube/swab container
  - Minimum information needed
    - Unique participant ID number
    - Date specimen collected
    - Exactly what specimen is (e.g., blood, wound swab)
- A specimen transport log form should be used, consisting of:
  - Place, date and time of collection shipment
  - Contents of shipment including participant ID numbers, specimen types and order of storage

*Specimen Transfer*

- Place absorbent material in sealed biohazard bags with specimens in case of sample leakage
- Put into recommended portable transport container. For samples collected into storage medium with refrigeration recommended (i.e., STGGB), store sealed bags in between ice cooler bricks
- Seal lid of portable container as instructed or with waterproof tape
- Label all containers clearly with:
  - Place, date, time of packing, and destination
  - Biohazard sticker. If no sticker, write it in big letters using black marker
- Make sure the courier knows what contents are, so they will not be left in a hot place and will be promptly delivered to the laboratory
- Specimens should reach the laboratory as soon as possible (≤10 days)

## Appendix 4: Comparisons of Advantages and Disadvantages of Active and Passive Surveillance

| **Advantages** | **Disadvantages** |
| --- | --- |
| ***Active surveillance*** |  |
| - Sensitive system that facilitates early detection of new cases - Higher case ascertainment rate - More accurate identification of cases - Ability to verify information in the case of missing data or suspected data entry errors - Data collected can be comprehensive and specific to the surveillance objectives - Can evaluate the quality and effectiveness of case-finding process, thus minimizing selection bias - Allows real-time analysis and ability to respond/modify approach to surveillance and care - Can promote disease awareness and good health practices | - Can be costly and resource-intensive - Requires dedicated surveillance staff and/or extensive training and upskilling - Can be demanding on surveillance sites |
| ***Passive surveillance*** |  |
| - Can be conducted retrospectively - Requires fewer resources than active surveillance - Can support real-time reporting | - Responsibility for reporting new cases lies with healthcare workers/laboratory staff; thus, it can be difficult to ensure compliance by healthcare providers and other reporters - Difficulties caused by lack of standardization in terms of case definitions and coding - Tends to under-report disease - Commonly associated with incompleteness of data recording or of microbiological studies - Often difficult to confirm data recording or entry errors retrospectively |

## Appendix 5: Administrative Health Databases

Administrative data from laboratory datasets and electronic medical records (EMRs) from primary healthcare and emergency departments covering whole communities can provide a timely and cost-effective surveillance option.

An important consideration when using EMRs to calculate disease estimates in a population is that the data are collected and coded as part of service delivery rather than for surveillance purposes. As such, EMRs are often prone to missing data on key fields and require the conversion of unstructured/narrative text, which can be resource-intensive and subjective. For EMRs that include or rely on free text, new methods in machine learning or deep learning could improve case identification.^2,3^ Data are limited to patients who attend health services, and are subject to variance in physician’s propensity to seek microbiological confirmation, which may be subject to bias (e.g., more severe infections, more clinically ambiguous, one not responding to treatment) and underestimate disease incidence. However, an advantage to administrative data is that, in well-established systems, data are collected systematically, well-structured and are often population-based. EMRs can form the basis of enhanced surveillance by using an additional data collection form to augment routinely collected data.

Routinely collected clinic data may be insufficient for evaluating potential cases against the full criteria required to meet surveillance case definitions, especially when microbiological testing is not routinely conducted or recorded. Further, the data may be insufficient for addressing other surveillance objectives, such as variant typing and antimicrobial susceptibility testing.

Despite these limitations, when used consistently across health services data, EMR can provide important surveillance data on disease burden, including populations at high risk, trends over time, geographic and seasonal patterns, and service utilization due to impetigo. The value of EMR data can be enhanced with the use of common case definitions, coding practices, and protocols for bacterial confirmation.

## Appendix 6: Definitions of Key Surveillance Terms

| **Syndromic surveillance** | Syndromic surveillance refers to the use of a clinical syndrome (a constellation of symptoms and signs) as the case definition for detection of suscept cases. Syndromic surveillance can be used for initial case detection, but laboratory confirmation should occur to increase the accuracy of the system.^4^ |
| --- | --- |
| **Active surveillance** | Active case detection means that designated public health surveillance staff are directly involved in detecting cases.^4^ |
| **Passive surveillance** | Passive case detection means that health facility staff detect and report cases to the public health system.^4^ |
| **Facility-based surveillance** | Facility-based surveillance is based on ascertainment of cases in persons who seek care at health facilities, including outpatient clinics, doctors’ offices, hospitals and emergency departments.^4^ |
| **Sentinel-site surveillance** | Sentinel-site surveillance refers to a system that captures cases at one or more specialized sites, such as hospitals, clinics, schools or pharmacies.^4^ |
| **Community-based surveillance** | Community-based surveillance is the systematic detection and reporting of events of public health significance within a community-by-community members. Community-based surveillance enables earlier detection of the disease of interest and captures illnesses in persons who do not seek care in a hospital.^5^ |
| **Population-based surveillance** | Population-based surveillance attempts to capture all cases in a well-defined catchment population (for example, the entire population of a country). |
| **Healthcare utilization surveys** | Healthcare utilization surveys seek to characterize the health care-seeking behavior of ill persons by describing where ill persons sought health care for their illnesses, and soliciting reasons for not seeking health care.^6^ |
| **Unique identifier** | Unique identifiers are unique numbers or numbers and letter combinations that are allocated to a specific individual person. |

## Appendix 7: Good Practice and Ethical Considerations

**Monitoring/Audit**

A systematic and independent audit of surveillance systems should be undertaken to ensure that surveillance and surveillance-related activities were conducted following the relevant surveillance protocol, SOPs, ethical guidelines, and regulatory requirement(s) established by local public health. Existing surveillance review tools can be modified to guide the investigation (e.g., WHO’s [*Tools for a surveillance review: Vaccine Preventable Diseases Surveillance Standards*](https://www.who.int/publications/m/item/vaccine-preventable-diseases-surveillance-standards-annex1)). Surveillance as part of a clinical study should adhere to the International Council for Harmonisation (ICH) Guidelines for Good Clinical Practice ^7^.

**Quality control and quality assurance**

A quality management plan should be written before the start of surveillance to establish and ensure the quality of processes, data, and documentation associated with surveillance activities. It encompasses both quality control (QC) and quality assurance (QA) activities.

Surveillance systems should develop SOPs to ensure confidentiality for all cases, ensure that clinical specimens and bacterial isolates obtained are not compromised by human and processing errors, validate data integrity, and maintain multiple layers of security. A SOP will ideally detail:

- Data storage. Including participants’ unique surveillance ID numbers in each respective dataset enables linkage to other datasets, such as hospital admissions, facilitating the capture of complications and ensuring that all personal identifying information is removed from research/surveillance datasets.
- Data evaluation for protocol compliance and source document accuracy.
- Document review (e.g., specimen tracking logs, questionnaires), who is responsible, and frequency.
- Who the responsible person is for addressing QA issues (correcting procedures that do not comply with the surveillance protocol) and QC issues (correcting errors in data entry).
- Staff training activities and processes for documenting surveillance staff training.
- Maintenance and strict adherence to surveillance delegation log (list of staff involved in the surveillance and their duties/roles).
- Clinical and laboratory SOPs and accreditation.
- Regular audits of surveillance data to ensure accuracy and completion.
- System for periodic and refresher training for surveillance team.

**Ethics of Surveillance**

The global network of WHO Collaborating Centres for Bioethics in collaboration with the U.S. Centers for Disease Control and Prevention developed ethical guidelines for public health surveillance, including common good, respect for persons, and good governance. The guidelines cover the (i) broad responsibility for undertaking surveillance and subjecting it to ethical scrutiny; (ii) obligation for ensuring appropriate protection and rights; (iii) considerations in making decisions about how to communicate and share surveillance data. The guidelines are available at <https://apps.who.int>. Countries should implement these guidelines and monitor them regularly. As appropriate, surveillance protocols should adhere to existing country-specific ethical guidelines.

## Appendix 8: **Invasive Strep A Infections: Clinical Syndromes and Related ICD-10 codes**

| **Type of Invasive Infection** | **Typical Body Sites From which Strep A is Cultured** | **Associated ICD-10 Codes*** |
| --- | --- | --- |
| Necrotizing fasciitis | Wound and blood | M72.6, NS*= nonspecific etiology |
| Streptococcal toxic shock syndrome | Blood, throat, and wound, if appropriate | A48.3**, *S aureus* |
| Septic shock | Blood | R65.2 NS; A40 NS |
| Puerperal sepsis | Blood and endometrium | O85 |
| Septic arthritis | Blood and joint fluid | M00.2X, NS |
| Streptococcal meningitis | Blood, cerebrospinal fluid | G00.2, NS = “streptococcal meningitis” |
| Cellulitis | Blood, sterilely obtained tissue aspirate/specimen | L03.XX, NS |
| Lymphangitis | Blood, sterilely obtained tissue aspirate/specimen | L03.91, NS; I89.1, NS |
| Osteomyelitis | Blood, sterilely obtained tissue aspirate/specimen | M86, NS |
| Empyema or pneumonia with effusion | Blood, pleural fluid | J86.0, NS “pyothorax with fistula”; J86.9, NS, “pyothorax without fistula” |
| Pneumonia | Blood, lung specimen (taken via bronchoalveolar lavage, bronchoscopy or open lung biopsy), and pleural fluid | J18.9, NS; J15.4, NS= “pneumonia due to other streptococci” |
| Bacteremia | Blood | R78.81, NS |
| Sepsis or septicemia | Blood | A40.9, NS = “Streptococcal sepsis”; A41.9, NS=“sepsis”P36.10, NS = “sepsis of newborn due to other unspecified streptococci” |
| Abscess | Blood, wound, sterilely obtained tissue aspirate/specimens | NS codes include: D73.3, E32.1, H60.0, H70.01, J36, J34.0, J39.x (x=0-2), J85, K11.3, K12.2, K61.x (x=0-4), K63.0, K65.1, K68.1, K75.0, L02.x (x=0-6, 8, 9) |
| Chorioamnionitis | Amniotic ﬂuid | NS codes: O41.12x (x=0-3, 9); P02.7 |
| Endocarditis | Blood, sterilely obtained vascular tissue aspirate/specimen | NS codes: I33.0, I33.9, I38, I39, I01.1 |
| Endometritis | Blood, sterilely obtained endometrium tissue aspirate/specimen | NS codes: O86.12, O86.8, O86.89 |
| Peritonitis | Blood and peritoneal fluid | NS codes: K65, N73.x (x=3-5), P78.1 |
| Pericarditis | Blood | NS codes: I01.0, I09.2, I30.x (x=1, 8, 9) |

ICD-10 Codes listed in Appendix 8 are current as of February 2021 and should be used as a guide only. Jurisdictions should be aware of country-specific modifications. Retrospective studies will need to identify ICD-10 (or ICD-9) codes used during the surveillance period.

X – additional number required: 0 = unspecified; 1–9 to specify site.

*NOTE: The positive predictive value of ICD-10 codes for invasive Strep A infections is often poor. Such codes are listed here as ‘NS’. NS: Not specific for Strep A. If surveillance relies on ICD diagnosis codes, a Strep A-specific code is required to classify the infection as invasive Strep A.

**A48.3 is for toxic shock syndrome due to *Staphylococcus aureus*. This ICD-10 code should only be accepted as a case of invasive Strep A infection if (A) Strep A or *S pyogenes* is isolated from blood, or (B) Strep A or *S pyogenes* is isolated from throat or wound, AND *S aureus* is NOT identified.

## Appendix 9: Variables fro Inclusion in Invasive Strep A Surveillance Datasets

| **Category of Variables** | **Required variables** | **Optional variables** |
| --- | --- | --- |
| **General** | - Participant unique ID number* - Date of enrolment | - Illness onset date |
| **Demographics** | - Age (in months if <1 year; otherwise in years) - Sex | - Date of birth - Race/ethnicity - Residential address |
| **Type of infection**  **(check all that apply)** | - Abscess (not skin) - Bacteremia (without focus) - Cellulitis (with bacteremia) - Chorioamnionitis - Empyema - Endocarditis - Endometritis - Meningitis - Necrotizing fasciitis - Osteomyelitis - Peritonitis - Pericarditis - Pneumonia - Puerperal sepsis - Septic shock/sepsis - Septic arthritis - Septic abortion - Streptococcal toxic shock syndrome - Cellulitis - Skin abscess - Other (list:______) |  |
| **Clinical conditions which could serve as potential portal of entry (non-sterile sites)** |  | - Acute skin lesion (including impetigo) - Chronic skin lesion - Scabies - Pharyngitis or scarlet fever in prior 7 days - Confirmed Strep A pharyngitis in prior 7 days |
| **Clinical risk factors for invasive infection** |  | - Underlying chronic illness - Underlying immunocompromising condition - Diabetes - Chronic lung disease - Chronic heart disease (e.g., heart failure) - Liver failure or cirrhosis - Active cancer (solid or hematologic) - Current alcohol addiction - Smoking - AIDS or CD4 count <200 - HIV infection - Injecting drug use - Hepatitis C - Connective tissue disorder (e.g., SLE) - Chronic steroid use - Pregnancy - Postpartum - Surgery or delivery of baby within past 7 days - Penetrating trauma within past 7 days - Varicella infection within prior 14 days |
| **Epidemiologic risk factors** |  | - Household crowding (number of people currently residing in household; number of bedrooms) - Attends daycare - Resident in long-term care facility - Prisoner/in jail - Homeless - Exposure to person with invasive Strep A infection in prior 7 days - Exposure to person with non-invasive Strep A infection in prior 7 days |
| **Severity of disease and outcome** |  | - Hospitalized - Death (within 30 days) - ICU (yes/no; number of days)** - Debridement - Amputation - Chronic kidney disease or need for dialysis - APACHE II score ≥30 within the first 7 days of culture isolation - Discharged to (home; rehab; other__) - Transferred to rehab unit after discharge - Other (_____*describe*) |
| **Treatment** |  | *Pre-hospitalization:*   - Antibiotics prior to admission: yes/no; if yes, β-lactam (penicillins, cephalosporins), macrolides, clindamycin, tetracycline, other   *During hospitalization*:   - Date of admission - Date of discharge - Admitted to ICU - Length of stay - Surgical intervention (amputation, debridement) - Intravenous immunoglobulin - Antibiotics (route of administration; name of antibiotic; duration; dose) - β-lactam alone, β-lactam + clindamycin, β-lactam + other, non- β-lactam |
| **Microbiology** | - Participant unique ID number* - Specimen unique IDǂ - Specimen collection date - Date and hour plate is inoculated - Date and hour plate is placed in incubator - Date(s) and hour(s) plate is read - Name of reporting laboratory - Laboratory ID (if present)* - Episode number (if repeated episodes from the same person are included) - Sterile site from which organism isolated: type (or source) of specimen (with choice list including but not limited to blood, bone, brain, joint, CSF, heart, kidney, liver, lung, lymph node, muscle/fascia/tendon, ovary, pancreas, pericardial fluid, peritoneal fluid, pleural fluid, spleen, vascular tissue, vitreous fluid) - Strep A (or *S pyogenes*) identified from sterile site: yes/no - Type of test - Care setting | - Strep A (or *S pyogenes*) identified from non-sterile site: yes/no - *If yes*,   - *non-sterile site from which organism isolated.*   - group identified (choose one): A, B, C, G or other - Anti-streptolysin O (ASO) and anti-DNAase B antibodies - Storage/transport identification number - Place/site of transfer of isolate for additional testing - Further testing ordered (e.g., *emm* typing, whole genome sequencing, anti-streptococcal antibody titers, speciation of large-colony β-hemolytic *Streptococcus*, antibiogram, etc.) |

*Enables linkage of laboratory data with participant, clinical and epidemiological data

**ICU = Intensive Care Unit; *Mark unknown if cultures are conducted post administration of antibiotics

ǂIf more than one specimen is taken, each specimen must have a unique ID number

Acute Physiology and Chronic Health Evaluation (APACHE) II score – Record the ‘worst’ measurement, defined as the measure that correlated with the most points.

## References

1. Breiman RF DJ, Facklam RR, et al. . Defining the group A streptococcal toxic shock syndrome: rationale and consensus definition. *JAMA*. 1993;269(3):390-391.

2. Solares JRA, Raimondi FED, Zhu Y, et al. Deep learning for electronic health records: A comparative review of multiple deep neural architectures. *Journal of Biomedical Informatics*. 2020;101:103337.

3. Wang Z, Shah AD, Tate AR, Denaxas S, Shawe-Taylor J, Hemingway H. Extracting diagnoses and investigation results from unstructured text in electronic health records by semi-supervised machine learning. *PLoS One*. 2012;7(1):e30412.

4. World Health Organization. Surveillance standards for vaccine-preventable diseases. 2018;

5. World Health Organization. A definition for community-based surveillance and a way forward: results of the WHO global technical meeting, France, 26 to 28 June 2018. *Eurosurveillance*. 2019;24(2)

6. Deutscher M, Van Beneden C, Burton D, et al. Putting surveillance data into context: the role of health care utilization surveys in understanding population burden of pneumonia in developing countries. *Journal of Epidemiology and Global Health*. 2012;2(2):73-81.

7. U.S. Department of Health and Human Services. *E6(R2) Good Clinical Practice: Integrated Addendum to ICH E6(R1) Guidance for Industry* Vol. 2021. 2018.
